# Emerging roles of histone modifications and HDACs in RNA splicing

**DOI:** 10.1093/nar/gkz292

**Published:** 2019-04-24

**Authors:** Raneen Rahhal, Edward Seto

**Affiliations:** George Washington Cancer Center, Department of Biochemistry & Molecular Medicine, George Washington University School of Medicine & Health Sciences, Washington, DC 20037, USA

## Abstract

Histone modifications and RNA splicing, two seemingly unrelated gene regulatory processes, greatly increase proteome diversity and profoundly influence normal as well as pathological eukaryotic cellular functions. Like many histone modifying enzymes, histone deacetylases (HDACs) play critical roles in governing cellular behaviors and are indispensable in numerous biological processes. While the association between RNA splicing and histone modifications is beginning to be recognized, a lack of knowledge exists regarding the role of HDACs in splicing. Recent studies however, reveal that HDACs interact with spliceosomal and ribonucleoprotein complexes, actively control the acetylation states of splicing-associated histone marks and splicing factors, and thereby unexpectedly could modulate splicing. Here, we review the role of histone/protein modifications and HDACs in RNA splicing and discuss the convergence of two parallel fields, which supports the argument that HDACs, and perhaps most histone modifying enzymes, are much more versatile and far more complicated than their initially proposed functions. Analogously, an HDAC-RNA splicing connection suggests that splicing is regulated by additional upstream factors and pathways yet to be defined or not fully characterized. Some human diseases share common underlying causes of aberrant HDACs and dysregulated RNA splicing and, thus, further support the potential link between HDACs and RNA splicing.

## INTRODUCTION

The human genome is comprised of ∼3.2 billion nucleotides, of which only 1.5% codes for proteins (1,2). Today we know that these non-coding regions, initially thought to be functionless ‘junk DNA’, consist of transposons, repeated sequences, pseudogenes and introns (3). However, it was back in the late 1970s that several labs, notably those of Phillip Sharp and Richard Roberts, independently revealed that introns, long stretches of non-coding DNA, separated protein-coding genes in eukaryotic cells (4,5). The subsequent finding of pre-mRNA splicing was surprising as it challenged the dogma of co-linearity between RNA and DNA, and ushered in a new era of molecular biology. Successively, introns were found to possess important biological functions and play crucial roles in regulating gene expression, and transcriptome diversification through alternative splicing.

**Figure 1.**
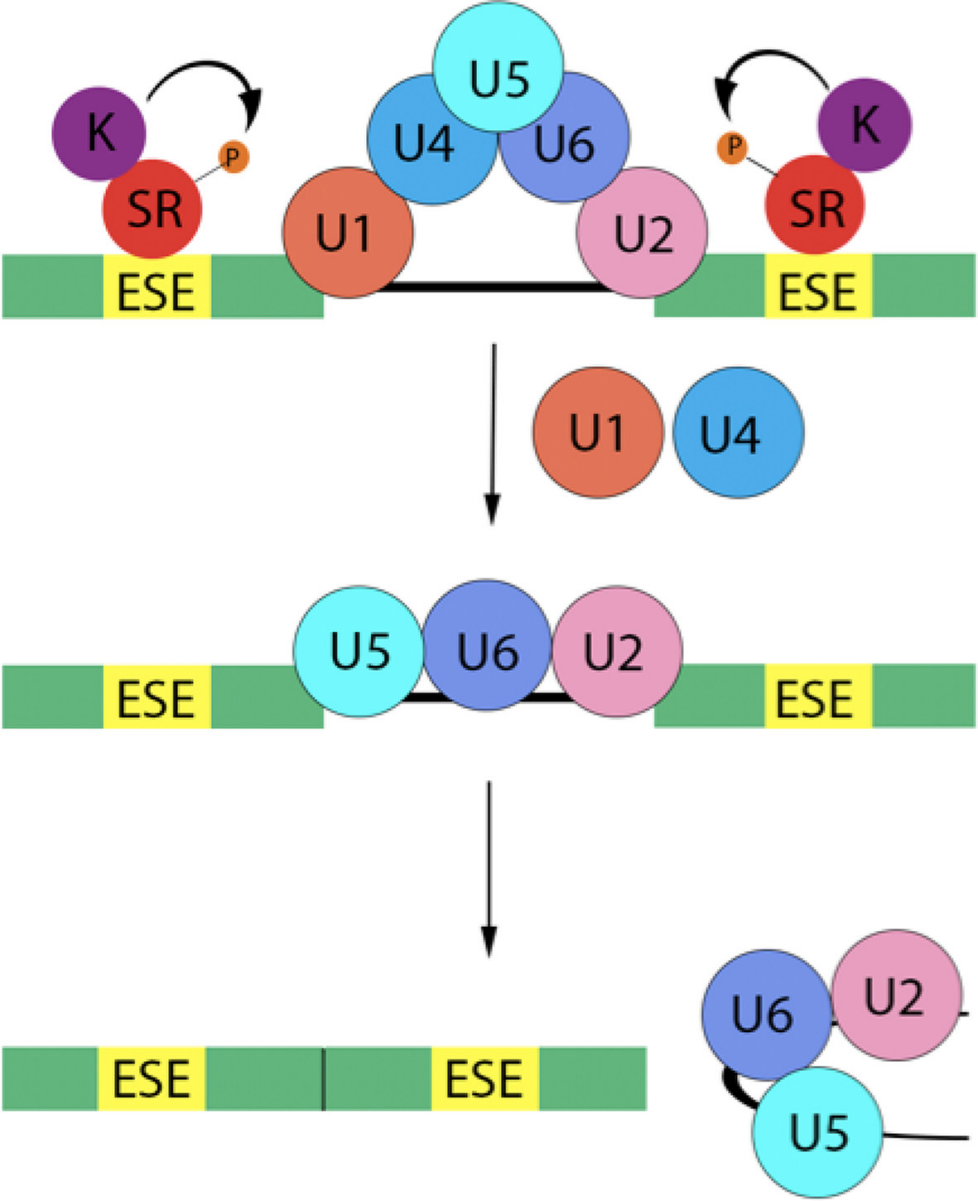
An illustration of spliceosome assembly and RNA splicing. Kinases (K) bind and phosphorylate serine-arginine (SR) proteins, which bind mRNA at exonic splicing enhancer (ESE) sites. This then recruits U1 and U2 proteins, signaling for the binding of U4/U6.U5 tri-snRNP. Upon release of the U1 and U4 protein complexes, the spliceosome is catalytically activated, removing introns and joining the remaining exons.

Alternative splicing is the process by which different regions of exons and introns are joined together to produce mature messenger RNA (mRNA) transcripts, which often lead to unique proteins or isoforms. This allows a single gene to code for numerous proteins. With over 90% of human genes undergoing alternative splicing, it is crucial to understand the mechanisms of alternative splicing to appreciate how this process, and ultimately, gene regulation is achieved (6,7). The main splicing machinery is the major spliceosome, a megadalton complex composed of five uridine-rich small nuclear RNAs (snRNAs)—U1, U2, U4, U5 and U6 (RNU1, RNU2, RNU4, RNU5 and RNU6)—as well as nearly 150 associated proteins, forming small nuclear ribonucleoproteins (snRNPs) (8). The spliceosome is signaled to assemble after positive-acting factors such as serine and arginine rich splicing factors (SRSFs), bind to *cis*-acting elements—exonic and intronic splicing enhancers. Similarly, spliceosome assembly can be inhibited by negative acting factors such as heterogeneous nuclear ribonucleoproteins (hnRNPs), binding to exonic or intronic splicing silencers. Since these silencing sites are located within close proximity to the enhancers, inhibitors binding to exonic or intronic splicing silencers, can sterically hinder activator proteins (9). Upon binding of SR proteins to splicing enhancers, the U1 and U2 protein complexes bind to the splice site and the branching site respectively. This then recruits the U4/U6.U5 tri-snRNP (SART1) to bind mRNA. However, it is not until the unwinding and release of U1 and U4 protein complexes that the spliceosome becomes catalytically active, allowing for the removal of introns followed by exon ligation (10) (Figure 1).

A fundamental focus within the complexity of RNA splicing is understanding the process by which exons and introns are identified in order to initiate spliceosomal assembly. Two basic models of early splice site recognition exist. In exon definition, each exon is recognized as an entity and joined to another similarly-recognized exon; the intron is removed as a result (11–13). At the molecular level, exon definition is thought to be engaged by U1 snRNP binding at the downstream 5′ splice site to promote U2 auxiliary factor (U2AF) recognition of the upstream 3′ splice site and subsequent U2 snRNP binding to the branch site across the exon. Exon splicing enhancers located in between the two splice sites recruit SR proteins to stabilize a protein-protein interaction network across the exon, forming a connection between U2 and U1 snRNPs (14). By contrast, in intron definition, the intron itself acts as the unit of recognition, and splicing machinery directly searches for two intron splice sites. Unlike the splicing machinery of exon definition which imposes a length constraint on exons but does not affect intron size, the splicing machinery of intron definition limits the size of introns, but not that of exons (15). In the human genome, the vast majority of exons are short and introns are long, thus most of the human splice sites are recognized across the exon. Conversely, in lower eukaryotes where genome architecture is characterized by small introns and large exons, the intron definition model is more likely predominant, where splice sites are initially paired across introns rather than exons. While splicing is a complex process, these two models provide a baseline for predicting the behavior of pre-mRNA.

### Co-transcriptional splicing

Early models presented the spliceosome as separate and distinct from transcriptional machineries, and the splicing event as a completely independent post-transcriptional process. However, evidence coupling the process of transcription and splicing quickly emerged. One of the first studies to demonstrate possible co-transcriptional splicing used electron microscopy of chromatin spreads from embryonic *Drosophila melanogaster* to show that intron looping was occurring in the presence of associated ribonucleoprotein complexes on transcripts joined to DNA, suggesting that splicing takes place prior to transcript release (16). Nearly a decade later, immunofluorescence was used to confirm that the localization of splicing factors at transcription sites occurred in intron-containing genes (17–19). More evidence emerged in recent years with the use of chromatin-RNA immunoprecipitation assays, showing that the recruitment of splicing factors, and splicing itself, occurs co-transcriptionally in yeast (20–22) and mammalian cells (23). Although the majority of splicing in yeast occurs post-transcriptionally, current data convincingly supports that many RNA splicing events in eukaryotic cells take place co-transcriptionally (24–28). Because post-translational modifications (PTMs) of histones profoundly regulate gene transcription, it is important to understand histone modifying enzymes such as histone/lysine deacetylases (HDACs/KDACs) which could co-localize, and exert their functions at splice sites.

### Alternative Splicing Regulation

**Figure 2.**
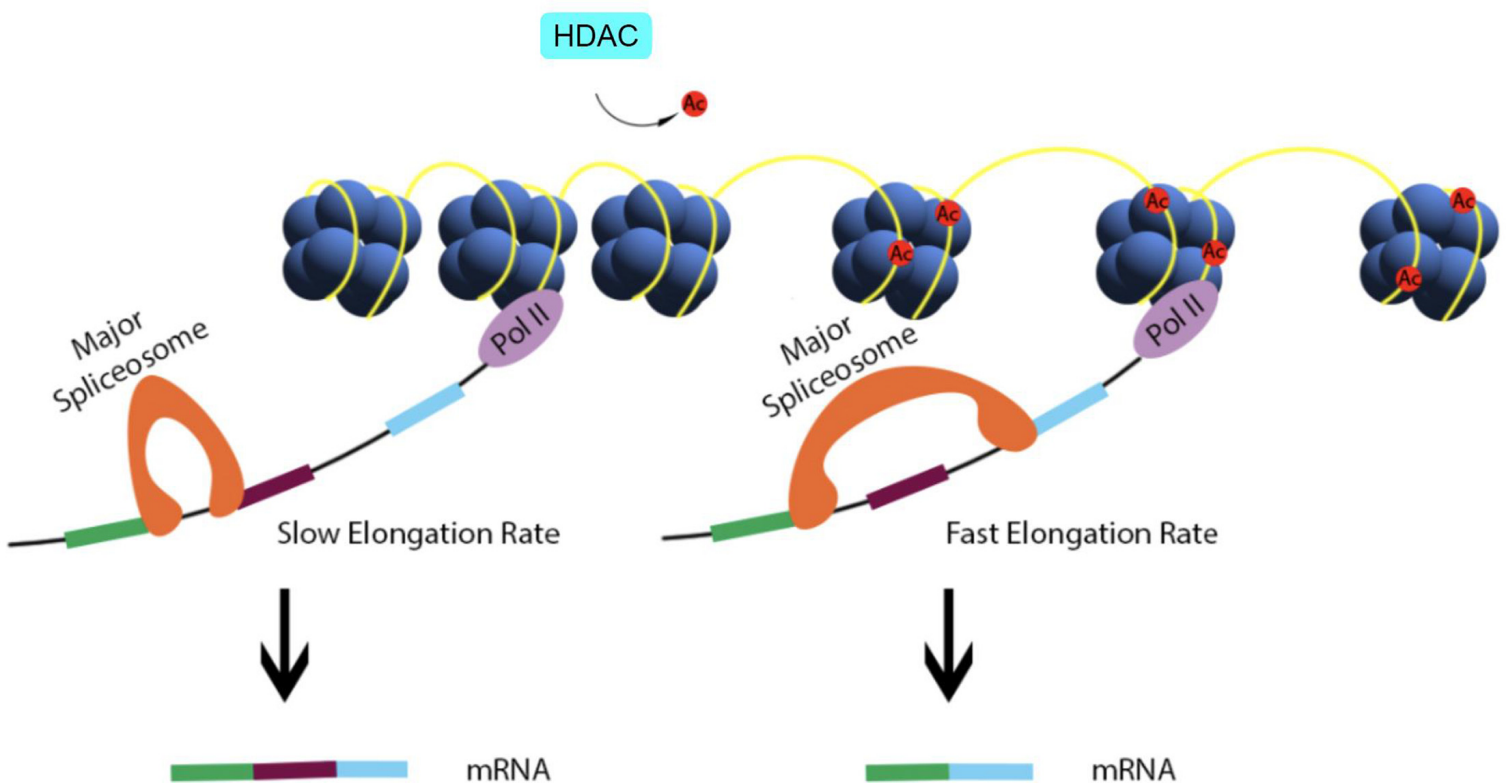
A schematic depicting the kinetic coupling system. During transcription, the major spliceosome is assembled and attached to the signaled splice sites. As illustrated in the left panel, when histones are deacetylated, chromatin are more tightly compacted, allowing for a slower elongation rate. As a result, splicing factors are recruited to a weak splice site, leading to exon inclusion. The right panel shows that histone acetylation creates a more open chromatin structure, allowing for a faster elongation rate, which promotes splicing factors recruitment to the strong splice site, resulting in exon exclusion.

Alternative splicing is a complex process which can be controlled via RNA-binding proteins (RBPs). RBP-dependent pathways rely on RBPs’ ability to bind pre-mRNA at specific sequences, controlling splicing patterns. RBPs modulate splicing in various ways, including controlling one another via cooperative or competitive binding to pre-mRNA (29). Although RBP-dependent alternative splicing represents the vast majority of studies on alternative splicing regulation, a new and exciting area in regulating alternative splicing is linked to chromatin structure and epigenetic modifications. In this case, no change in RBP expression level or localization is needed to result in a change of splicing pattern. Two mechanisms have been proposed that implicate epigenetic components, such as chromatin structure and histone modifications, to alternative splicing regulation: kinetic coupling and chromatin-splicing adaptor systems. The kinetic coupling model suggests a competitive nature between splicing and the transcriptional elongation rate, whereby a faster elongation rate will favor the recruitment of splicing factors to the strong splice site, resulting in exon skipping. In contrast, a slower elongation rate will recruit splicing factors to the weak upstream splice site, resulting in exon inclusion (Figure 2). The chromatin-splicing adaptor system proposes that chromatin remodeling proteins have the ability to recruit splicing factors to transcriptional sites or to sites of specific exons, directly influencing exon inclusion and exclusion (30). While these models are non-mutually exclusive and act independently, they are both affected by histone PTMs.

PTMs are crucial to the formation and function of mature protein products, and can occur at the protein's N- or C-terminal, as well as at amino acid side chains. One of the most common PTMs is the reversible addition of an acetyl moiety to the ϵ-amino lysine residue of proteins. This modification is controlled by histone/lysine acetyltransferases (HATs/KATs) and HDACs, which act to add and remove acetyl groups from lysine residues, respectively. Studies have shown that histone deacetylation plays a fundamental role in modulating gene transcription and chromatin structure (31–33), in addition to a myriad of cellular processes (34,35). However, a new and exciting area worth exploring is the potential of HDACs in regulating alternative splicing by modulating kinetic coupling, the chromatin-splicing adaptor system, or via altering other regulatory mechanisms.

### HDACs

Histone modifications, acetylation/deacetylation in particular, were first described more than a decade prior to the discovery of RNA splicing. In stark contrast to the exciting finding and immediate explosion in the RNA splicing field in the 1970s, histone deacetylation research took off slowly and was dormant for a long period. Early studies pioneered by Vincent Allfrey showed that relatively minor histone modifications could greatly influence the rate of transcription (36). These modifications, especially acetylation and deacetylation, were implicated in providing a dynamic and reversible mechanism for turning RNA synthesis on or off at different times and at different chromosomal loci.

HDACs were originally identified as enzymes that catalyze the removal of acetyl moieties from the ϵ-amino groups of conserved lysine residues in the amino terminal tail of histones. The removal of this modification strengthens histone–DNA interactions and/or generates specific docking surfaces for proteins that regulate chromatin folding and/or transcription. Transcriptionally active chromatin is generally associated with histone hyperacetylation, which could be responsible for increasing the accessibility of nucleosomal DNA to transcription factors. Conversely, hypoacetylation of histones correlates with transcriptional silencing. Soon after the report of HDAC enzymatic activity, many attempts were made to purify homogeneous HDACs using conventional chromatography without success (37). Despite early awareness of the potential biological importance of HDACs, it took many years before two bona fide HDACs were finally isolated and cloned in 1996, spurring an intense interest in this field (38). Today, most discussions on eukaryotic transcriptional repression refer to some aspects of histone deacetylation and HDACs. Results from numerous studies validate the prediction that HDACs play crucial roles in gene transcription and affect many eukaryotic biological processes that involve chromatin (39). In addition to its effect on transcription, HDACs also have important roles in chromatin assembly, recombination, and chromosome segregation. Multiple studies have revealed that many HDACs possess the ability to deacetylate not only histones, but also non-histone protein substrates, suggesting a role in non-chromatin/transcription related biological processes (40). Most interestingly, recent studies reveal that some HDACs possess other enzymatic activities in addition to deacetylation (41,42).

To date, 18 human HDAC enzymes have been identified and are categorized into four classes based on sequence homology. Class I HDACs share sequence similarities with the yeast Rpd3 protein, and are comprised of HDAC1, HDAC2, HDAC3 and HDAC8. Class II HDACs share sequence similarities with the yeast Hda1 protein, and are divided into subclasses IIa and IIb, which are comprised of HDAC4, HDAC5, HDAC7 and HDAC9, and HDAC6 and HDAC10, respectively. Both class I and II HDACs share sequence homology with the yeast Hos proteins. Class III HDACs share sequence similarities with the yeast Sir2 protein, and are comprised of SIRT1, SIRT2, SIRT3, SIRT4, SIRT5, SIRT6, and SIRT7. Class IV, which is comprised of only HDAC11, shares sequence similarities to both Class I and II HDACs (31,43,44).

Class I, II, and IV HDACs belong to the classical HDAC family, sharing not only sequence similarities, but also similarities in tertiary structures and functions. The catalytic activities of these HDACs are metal-dependent, whereby the acetamide bond in acetylated lysines undergo hydrolysis. Class III HDACs, however, belong to the Sir2 regulator family, and function via an NAD^+^-dependent mechanism. A byproduct of this nucleophilic reaction, nicotinamide, acts as sirtuin inhibitor (31). While increasing evidence suggests that HDACs play a role in RNA splicing, currently there is no conclusive evidence in the literature that the involvement of HDACs in splicing is specific and limited to a certain family, class, or subset of HDACs.

## HISTONE MODIFICATIONS IN RNA SPLICING

Eukaryotic proteins are subjected to over 200 documented types of post-translational modifications, which greatly increases protein diversity (45). Histones, like most eukaryotic proteins undergo many different types of PTMs including acetylation, methylation, phosphorylation, and ubiquitination. Human histones contain ∼130 known post-translationally modified sites, often referred to as ‘marks’ (46). Many of these marks are widely established as regulators of gene expression by altering chromatin structure or by recruiting histone modifiers, to activate or repress transcription. Less known, but perhaps just as important, is how these modifications/marks control gene expression via regulating RNA splicing. Here we discuss three signature histone modification marks that may affect splice site choice and serve as splicing regulators.

### H3K36 methylation

Histone methylation is equally as important as histone acetylation in altering histone–DNA interactions and affecting gene transcription. A lot of our knowledge concerning the role of HDACs in RNA splicing, stems from our knowledge of histone methylation and splicing. Histones are methylated by histone methyltransferases (HMT) which catalyze the addition of one, two, or three methyl groups to arginine or lysine residues. There are two main categories of HMTs, arginine-specific and lysine-specific; the latter of which can be subdivided into SET (Su[var]3-9, Enhancer of zeste and Trithorax) domain-containing HMT and non-SET domain-containing HMT.

Common sites of histone methylation associated with transcriptional activation include H3K4, H3K36, H3K48 and H3K79, while common methylation sites for transcriptional repression include H3K9 and H3K27 (47). Histone H3K36 is one the most common sites that undergoes PTMs; the role of methylation in RNA splicing is best exemplified in trimethylated H3K36 (H3K36me3). In yeast, a single H3K36 methyltransferase, Set2 (KMT3), catalyzes all three states of H3K36 methylation. Mammalian cells contain at least eight H3K36 methyltransferases: NSD1 (KMT3B), NSD2 (KMT3G), NSD3 (KMT3F), SETD2 (KMT3A), SETD3, SETMAR, SMYD2 (KMT3C) and ASH1L (KMT2H). Of these enzymes, only SETD2 can catalyze H3K36 trimethylation, whereas the other seven enzymes are restricted to H3K36 mono- and/or dimethylation (48). H3K36 demethylases include JHDM1B (KDM2B) and JMJD2 (KDM4A) (49,50).

Although H3K36 methylation is generally associated with transcriptional activation, it has also been implicated in diverse processes, including dosage compensation, transcriptional repression, DNA repair and recombination, and alternative splicing. For example, dimethylated H3K36 (H3K36me2) plays a role in recruiting early repair factors such as Ku70 (XRCC6) and Nbs1 (NBN), at the site of double strand breaks (51). H3K36me3 plays an important role not only in transcription, but also in alternative splicing. In yeast, H3K36me3 is enriched in the body of actively transcribed genes, and is found at significantly lower densities within introns, compared to surrounding exons (52). Other studies suggest that in yeast, this mark recruits and binds to HDACs, which deacetylate neighboring histones in order to prevent runaway transcription (53–55).

In mammalian cells, studies have shown a correlation among H3K36me3, genomic DNA methylation, and cryptic transcription. Silencing of DNA methyltransferase 3 beta (DNMT3B) in mouse embryonic stem cell lines revealed a reduction in DNA methylation and a concomitant trimethylation of H3K36. Silencing SETD2, an H3K36me3 specific histone methyltransferase, in mouse *Dnmt3b*^−/−^ and wild-type embryonic stem cells, resulted in a decrease in H3K36me3 in both cell lines, in addition to cryptic transcription. Interestingly, there was a loss of *Dnmt3b* intragenic DNA binding in the wild-type cells, suggesting an association between the methyltransferases. DECAP-seq revealed an increase in transcription start sites (TSSs) on gene bodies of *SetD2* knockdown cells, compared to control cells. Approximately 75% of these TSSs were unique to the *SetD2* knockdown, while over 50% were identical to the ones seen in *Dnmt3b* knockout (KO) cells. Therefore, it is fair to assume that there is an association between genomic DNA methylation, and histone methylation in the regulation of transcription, though the exact mechanism has yet to be established (56). The importance of SETD2 has been shown in other studies, where silencing *Setd2* in Apc^min/+^ mice, altered the alternative splicing of ∼700 genes (57).

Because H3K36me3 is found at low levels at the promoter and is enriched at transcribed regions of genes (57,58), particularly over exons in poorly expressed genes (52), it is believed to modulate exon definition by regulating the rate of RNA polymerase II elongation (59). Its ability to influence splicing is seen in fibroblast growth factor receptor 2 (FGFR2). Two major splice variants of this gene exist: *FGFR2-IIb* expressed in epithelial cells, and *FGFR2-IIIc* expressed in mesenchymal cells. Interestingly, H3K36me3 is only enriched in mesenchymal cells, where it is recognized by MORF (KAT6B; MYST4)-related gene 15 (MRG15; MORF4L1)). MRG15, then recruits and binds polypyrimidine tract-binding protein (PTB), a splicing repressor which interacts with an intronic splicing silencer surrounding exon IIIb, to repress its inclusion in mesenchymal cells (60). Additionally, H3K36me3 has also been shown to be associated with PSIP1/p52 which are both enriched in highly expressed genes, such as versician (Vcan). Here, PSIP1 (p52) binds to H3K36me3, recruiting SRSF1 (ASF1; SF2), a splicing factor which binds to exonic splicing enhancers in order to promote exon inclusion (61). Dysregulated H3K36 methylation can lead to a range of human diseases, underscoring the importance of this modification.

### H3K9 Methylation

Histone H3K9 methylation typically silences gene transcription and is a mark of heterochromatin. In mammalian cells, the major H3K9 methyltransferases are SUV39H1 (KMT1A), SUV39H2 (KMT1B), SETDB1 (KMT1E), G9A (EHMT2; KMT1C), and the PR/SET domain (PRDM) family (62). Each of these HMTs possesses different catalytic activities and target different genes to achieve diverse cellular functions. Demethylation of H3K9 is catalyzed by LSD1 (KDM1A) and several Jumonji Domain-Containing demethylases including JMJD1B (KDM3B), JMJD2A (KDM4A), JMJD2B (KDM4B), JMJD2C (KDM4C) and JMJD2E (KDM4E) (63–65).

Like H3K36me3, it is well established that H3K9 methylation affects alternative splicing. Histone methylation marks are crucial in identifying splice sites in pools of exons. This can be seen with CD44, a cell surface glycoprotein involved in hematopoiesis, migration, lymphocyte activation, cell adhesion, and cell-cell interactions. Many isoforms of this protein exist, all of which are comprised of two series of constitutive exons (exons 1–5, and exons 16–19), and ten variable exons (exons 6–15). Studies have shown that the regions of these variable exons are enriched in H3K9me3 marks which are recognized by the heterochromatin 1 protein HP1γ (CBX3). This interaction leads to a reduced local transcriptional elongation rate and therefore, inclusion of variable exons in the mature mRNA (66). Similarly, H3K9me2 enrichment in fibronectin (*FN1*) was found to recruit HP1α (CBX5), also leading to a reduced elongation rate, and inclusion of exons (67).

### H3K9 acetylation

While methylated H3K9 can silence gene transcription, acetylated histone H3K9 can activate gene transcription. H3K9ac is particularly important because it is highly correlated with active promoters (68). H3K9 is predominantly acetylated by the well-characterized PCAF/GCN5 (KAT2B/KAT2A) class of lysine acetyltransferase (69). In addition, p300 (EP300; KAT3B) has also been reported to acetylate H3K9 (70).

Like histone methylation, histone acetylation can also alter alternative splicing (71). For example, this can be seen in the regulation of neuronal cell adhesion molecule (NCAM; NCAM1; CD56) expression. NCAM is a glycoprotein which plays a role in the development of the central nervous system. In addition, NCAM has a causal role in cancer aggressiveness, may promote cancer progression, and plays a role in tumor metastasis. While there are twenty-seven known spliced variants of *NCAM*, there are two main isoforms (NCAM-140 kDa and NCAM-180 kDa) that differ only in their cytoplasmic domain due to the selective exclusion of exon 18. Upon depolarization, H3K9 hyperacetylation is induced and restricted to regions surrounding exon 18. This open chromatin structure results in a faster local RNA polymerase II elongation rate, which leads to exon skipping (72). Many HDACs including HDAC3, HDAC11, SIRT1, and SIRT6 have been reported to deacetylate acetylated histone H3K9 (H3K9ac) (31). Further work will be required to determine if these HDACs control RNA splicing through the deacetylation of H3K9.

## HDACS’ ROLE IN RNA SPLICING

### HDACs’ Impact on Splicing via Kinetic Coupling

Although the role of HDACs in alternative splicing remains unclear, there is strong evidence that an association between the two exists. Splicing-sensitive exon arrays of HeLa cells treated with sodium butyrate (NaB), a broad spectrum non-selective HDAC inhibitor, demonstrated a change in the splicing pattern of ∼700 genes (73). Treatment with this HDAC inhibitor, however, did not affect the level of phosphorylated SR proteins. Although the mechanism is unclear and the effect might be indirect, there seems to be a correlation between HDAC inhibition and exon exclusion. Upon *FN1* analysis post-NaB treatment, there was a decrease in H3 acetylation at the promoter, but an overall increase in H4 acetylation over the gene body. Interestingly, H4 acetylation was specifically prominent at exon 25, also known as Extra Domain B (EDB), which resulted in EDB exclusion. Further investigation identified that HDAC1, but not HDAC2 knockdowns, resulted in EDB skipping (73). This suggests a potential regulatory role of HDAC1 in the splicing of *FN1*.

A similar outcome is seen in *MCL1*, a gene that encodes two proteins: MCL1-long form (MCL1_L_) and MCL1-short form (MCL1_S_). MCL1_L_ is upregulated in cancer due to its anti-apoptotic nature. The difference in these isoforms is a result of the exclusion of exon 2. Inhibition of HDACs using trichostatin A (TSA), apicidin, or NaB, all led to an increase in H4 acetylation, specifically at exon 2, resulting in exon 2 skipping, forming MCL1_S_. Correspondingly, HDAC1 and HDAC2 knockdown caused exon 2 skipping and a preferential increase in MCL1_S_. The same effect is seen among SRSF1 silencing (74). HDAC inhibition in the human colorectal carcinoma HCT 116 and breast cancer MDA-MB 231 cell lines also resulted in an overall increase in H3 acetylation, as well as an increase in H3K4me3 along exon 2 of *MCL1*. In addition, there was a reduction in the association of HDAC1, HDAC2, and SRSF1 with exon 2, but an increase in the acetyltransferase PCAF. These results, however, were not consistent with those seen in MCF7 breast cancer cells where HDAC inhibition had little to no effect on H3K4me3, as well as on HDAC2 and SRSF1 occupancy along exon 2. This could be due to the higher levels of KDM5B (JARID1B), an H3K4me3 demethylase, and to the lower levels of KMT2G (SETD1B) and KMT2A (MLL), specific H3K4 methyltransferases, in MCF7 cells compared to MDA-MB 231 cells (75).

Coimmunoprecipitation assays determined that HDAC1 and hypophosphorylated HDAC2, coimmunoprecipitate with SRSF1. Additionally, among other proteins, HDAC1 and HDAC2 co-chromatin immunoprecipitated with SRSF1 along the gene body of *MCL1*. 12-*O*-tetradecanoylphorbol-13-acetate (TPA) stimulation increased the co-occupancy of both HDAC1 and HDAC2 with SRSF1 at exons 1 and 3 of *MCL1* (74). This indicates that HDAC1 or HDAC2, together with SRSF1, play a role in regulating the splicing of *MCL1*, though the mechanism has yet to be determined. Studies have shown that although SRSF1 is acetylated (76), HDAC inhibitors had no impact on this acetylation, nor SRSF1 expression levels (74). No differences in HDAC2 and acetylated histone distribution over the *MCL1* gene was detected after SRSF1 knockdown. This suggests that SRSF1 regulates the splicing of MCL1 independently of histone acetylation. However, HDAC inhibition did decrease the association of both SRSF1 with the *MCL1* gene, and of SRSF5 (SFRS5) with the *FN1* gene (73,74). It is important to note that SRSF1 is not the sole regulatory factor of MCL1. In a separate study, treatment of lung carcinoma A549 and H1299 cells with meayamycin B, an SF3B1 inhibitor, led to an increase in MCL1_s_ (77).

These two examples of single gene studies with *FN1* and *MCL1* demonstrate that histone deacetylation and HDACs modulate alternative splicing patterns. Although it is not clear whether this is a direct or indirect effect, it does provide broader insights into mechanistic understandings of how histone modifications, deacetylation and HDACs in particular, might influence RNA splicing. These findings have profound implications linking HDACs and splicing regulation and beg for more thorough investigations in this exciting area.

HDACs may play a further role in regulating alternative splicing by modulating the stability of hnRNPs. hnRNPs are a family of RNA-protein complexes which play critical roles influencing RNA metabolism, including alternative splicing. According to the protein modification database, 30 out of 35 hnRNPs contain at least one lysine residue that can be both acetylated and ubiquitinated. Among these is hnRNP F, which consists of three known lysines that are targets of acetylation and ubiquitination: K87, K98, and K224. Wild-type hnRNP F protein was found to be stabilized in the presence of TSA or lactacystin, a proteasome inhibitor. However, individual lysine to arginine mutants of K87, K98, and K224, do not experience a further increase in hnRNP F expression levels after TSA treatment. This concept is seen in other hnRNPs, such as in hnRNP A1, I and L, where the degradation of these K^ac/ub^ containing proteins after cycloheximide treatment, is rescued in the presence of TSA. Together, these data suggest that the stability of hnRNPs, which are key players in alternative splicing, is controlled by both acetylation and ubiquitination, where crosstalk between these two pathways may be common (78).

An important regulator of alternative splicing is calcium, which has been shown to change splicing patterns in both neuronal cells and cardiomyocytes. In one study, an exon array identified over 5000 genes whose splicing was altered due to an increase in calcium levels (79). In another study, mouse cardiomyocytes were treated with KCl to depolarize and, hence, increase intracellular calcium levels (80). After 24 h of treatment, there was a drastic decrease in neurofibromin 1 (*Nf1*) exon 23a inclusion from 72% to 9%. A decrease in exon inclusion was also seen in *kinectin 1* (*Ktn1*) exon 36, *ankyrin 2* (*Ank2*) exon 21, *Enah* exon5, and *myocyte enhancer factor 2A* (*Mef 2A*) exon 11. Interestingly, these changes in splicing patterns were reversed after the cells were switched to normal (KCl free) media. KCl treatment specifically reduced the level of nuclear class II HDACs, and increased H3 and H4 acetylation 2.5-fold. Additionally, KCl treatment led to a reduction in RNA polymerase II occupancy with *Nf1* and *Ktn1* (two genes with calcium-sensitive exons), as well as a faster transcriptional elongation rate. Together these data support the kinetic coupling model, whereby an increase in calcium levels increases histone hyperacetylation, which leads to a faster elongation rate and hence, exon skipping.

### Non-histone HDAC substrates in splicing

After the discovery of HDACs, many laboratories initially focused on understanding how HDACs remove acetyl groups from histones and the consequences of deacetylated histones on chromatin and transcription. Later studies however, showed that in addition to histones there is an abundant number of non-histone proteins that are targets of HDACs (40). Not surprisingly, pre-mRNA encoding many non-histone HDAC substrates are subjected to regulation by alternative splicing. However, unlike the coupling of transcription and RNA splicing, there is no reason to believe that RNA splicing, which takes place in the nucleus, can be coupled to translation/PTM/deacetylation, which occurs in the cytoplasm. Furthermore, although transcription and translation are coupled in prokaryotic cells, and prokaryotes possess self-splicing abilities, they lack splicing machinery. Interestingly though, protein acetylation is widespread in bacteria (81). Therefore, it is nevertheless intriguing to speculate that HDACs could multi-task by regulating the gene expression of many non-histone proteins through modulating RNA splicing and subsequently modifying those same proteins through post-translational deacetylation.

The p53 protein (TP53) is an example of a non-histone substrate that is regulated by HDACs potentially at both the RNA splicing and at the PTM level. p53 is an important cell cycle regulator and a tumor suppressor. It is one of the earliest non-histone HDAC substrates identified (82–84). The *p53* gene is comprised of 11 exons which undergo splicing to generate twelve known isoforms. Among these isoforms, is p53β which is brought about by the inclusion of intron 9 (i9), also known as exon 9b. Overexpression of p53β is a driver of cellular senescence (85). To better understand the modulation of full length p53 (p53) and p53β, it is important to study factors that regulate the splicing of *p53* RNA. Separate knockdown experiments of SRSF1 and SRSF3 (SFRS3) promoted the upregulation of p53β at the mRNA and protein level, as well as its activity, which was seen in an increase in SA-beta-galactosidase (85,86). In human fibroblasts, where SRSF3 was knocked-down, the increase in p53β was accompanied by an increase in p53 phosphorylation at serine 15, but not at the p53 protein level. Phosphorylation at this site is associated with p53 activation (85). This could explain the enhanced p53 transcriptional activity on the *p21* (*CDKN1A*; CIP1; WAF1) and *Bax* promoters observed after SRSF1 silencing in MCF7 cells (85).

**Figure 3.**
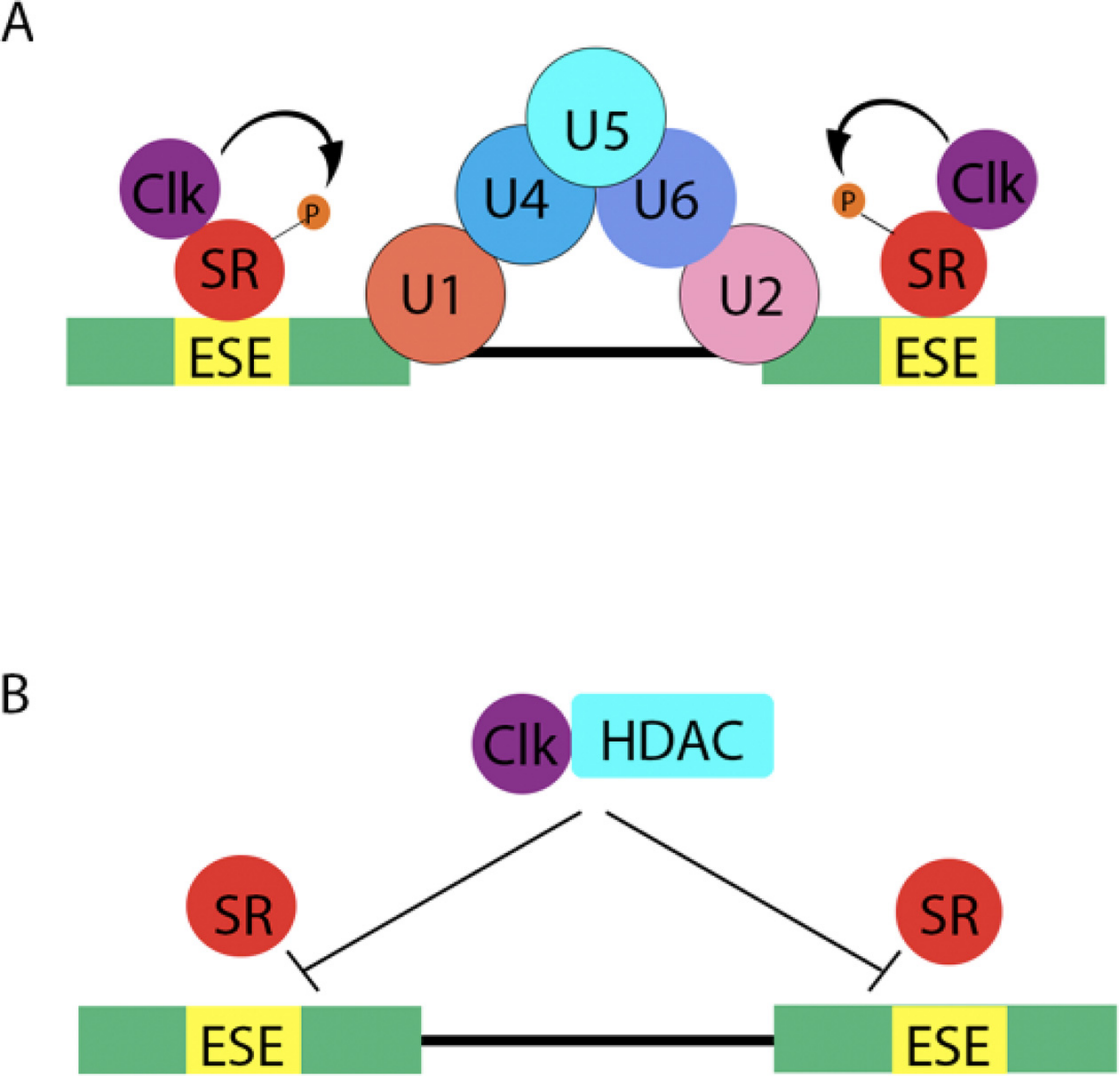
A model of spliceosome assembly (**A**) in the absence of HDACs, where Clk and other kinases (not shown) phosphorylate SR proteins, enabling them to bind to exonic splicing enhancers (ESEs), triggering a cascade of proteins to form the major spliceosome. (**B**) In the presence of HDACs, Clks are unable to phosphorylate SR proteins, inhibiting spliceosomal assembly.

Previous studies have shown that SR splicing factors must be phosphorylated in order to bind RNA (87–94). Dephosphorylation of SRSF3 by calf intestine phosphatase treatment in human fibroblasts, inhibited SRSF3 from binding to RNA (85). Accordingly, treatment with TG003, a Clk inhibitor, led to the dephosphorylation of SRSF1. When MCF7 cells were treated with TG003, there was an increased expression of p53β and a decrease of p53 (86), which most likely resulted from the inability of non-phosphorylated SRSF1 to bind mRNA. Taken together, it is fair to speculate that the coimmunoprecipitation of HDACs and SR proteins is not necessarily due to a direct interaction, but because they both interact with another protein or complex of proteins. Conceivably, CDC-like kinases’ (Clks’) and other kinases’ interaction with HDACs, inhibits its ability to phosphorylate the SR proteins (illustrated in Figure 3). As a consequence, SR proteins are incapable of binding to exonic splicing enhancers, which would normally trigger spliceosome assembly.

**Figure 4.**
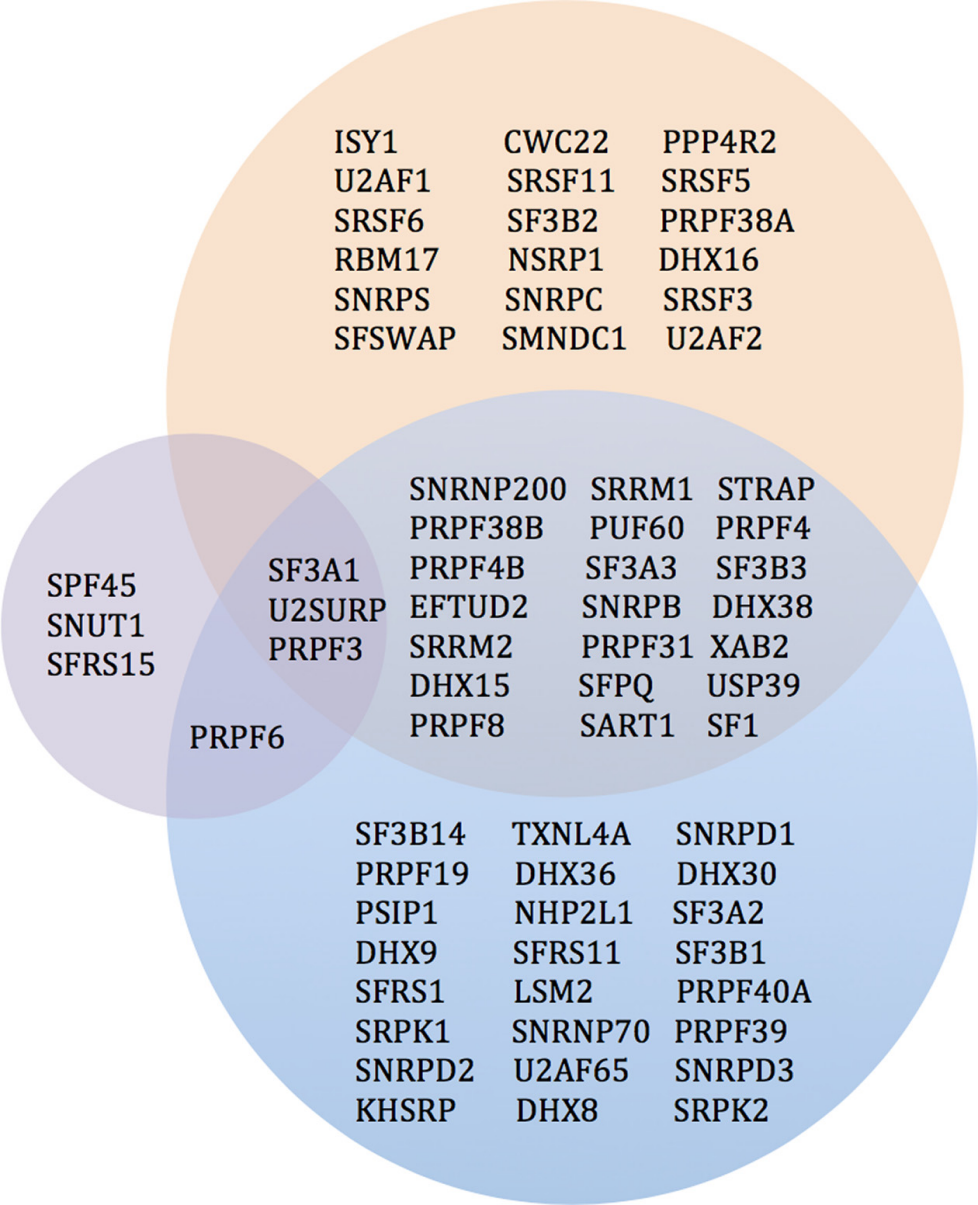
Splicing-associated HDAC-substrates. Comparison of results from three independent studies of splicing-associated proteins that are hyperacetylated with HDAC inhibitors treatment, or SIRT1 KO, are shown. SILAC data from Scholz *et al.* (97) of splicing-associated proteins that were hyperacetylated in HeLa cells due to treatment with HDAC inhibitors is shown in orange (upper circle). Data of such proteins from Sirt1 KO MEF cells are shown in purple (lower left) and blue (lower right) from Peng *et al.* (96), and Chen *et al.* (95) respectively.

Perhaps the most convincing evidence that HDACs play a key role in RNA splicing comes from several studies to identify non-histone HDAC substrates with stable isotope labeling in amino acids in cell culture (SILAC), an unbiased method that relies on mass spectrometry to detect protein changes (95–97). In these experiments, comparisons were made between HDAC inhibitors, primarily nicotinamide (NAB), treated versus non-treated cells or between HDAC KO cells, primarily deleted SIRT1, versus wildtype cells. By carefully analyzing the results from three different groups, we devised a list of non-histone SIRT1 substrates that were previously known to play a role in RNA splicing (Figure 4).

By comparing the splicing-associated proteins that showed hyperacetylation under SIRT1 inhibitor treatment or *Sirt1* KO, we identified three proteins that overlapped in three independent studies: PRPF3, SF3A1 and U2SURP. All three proteins are part of the splicing machinery. PRPF3 is part of the U4/U6.U5 tri-snRNP complex, while SF3A1 is a subunit of the SF3A1 splicing factor complex, which is important for the conversion of U2 snRNP from an inactive to an active form. Lastly, U2SURP is a splicing factor which belongs to the SR family, but its function remains unknown. These data strongly support a potentially important role of HDACs in deacetylation of non-histone proteins including splicing factors, and reveal SIRT1 in particular, in the RNA splicing pathway.

### HDAC and RNA splicing factor protein–protein interactions

Proteomics and bioinformatics data have further strengthened the link between HDACs and RNA splicing. The first global HDAC-protein interaction network for all 11 classical HDACs was established by Ileana Cristea. By generating EGFP-tagged CEM T-cells, each expressing a specific HDAC, the Cristea lab identified protein interaction via 1D nanoliquid chromatography/mass spectrometry. In order to assess the specific interactions, the Significance Analysis of INTeractions (SAINT) algorithm was employed, which resulted in the identification of over 200 previously unreported HDAC-protein interactions. Of these proteins, 124 were found to interact with HDAC11, many playing roles in RNA editing and processing. The association between HDAC11 and several proteins, notably SMN1, was validated by reciprocal isolations using an HDAC11 specific antibody. To further examine this interaction, HDAC11 was silenced in wild-type CEM T-cells. Similar to previous studies that silenced SMN1, HDAC11 knockdown resulted in the mis-splicing of *ATXN10* mRNA (98). While functional analysis of this network of interactions has only been touched upon, these findings along with the data from the SILAC experiments provide us with a strong foundation that a regulatory role exists between HDACs, particularly HDAC11, in RNA metabolism and splicing.

**Figure 5.**
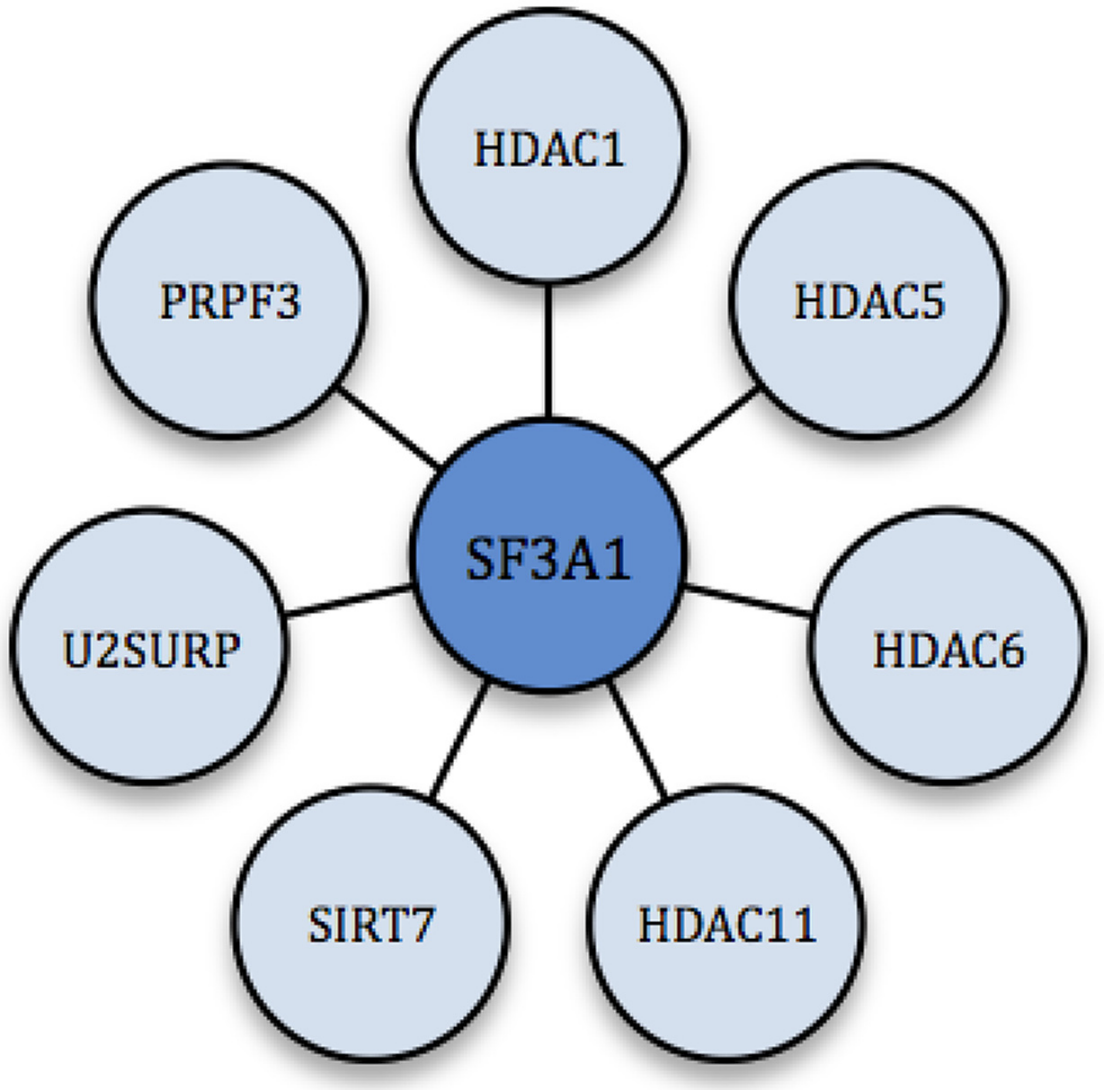
An illustration depicting the overlap of interactions among splicing-associated proteins and HDACs presented in Table 1.

To further investigate the relationship between HDACs and RNA splicing, we analyzed several public databases, including BioGRID and HRPD, to identify splicing-associated proteins that interact with HDACs (Table 1). Interestingly, some of these HDAC-interacting proteins also exhibited an increase in acetylation after HDAC inhibition or SIRT1 KO in non-histone substrate screens with SILAC (Figure 4). These results, together with previous findings that HDACs complex with proteins that regulate RNA splicing in a global protein interaction network for all classical HDACs (98), further supports the idea that HDACs play an important role in regulating RNA splicing. Furthermore, several HDACs as well as PRPF3 and U2SURP have been found to interact with SF3A1 (Figure 5). This indicates that SF3A1 could be a versatile protein that potentially plays an important role in HDAC-splicing regulation.

**Table 1.** Splicing-associated HDAC-interacting proteins including splicing factors, snRNPs, pre-mRNA processing factors, U4/U6.U5 tri-snRNP associated proteins, and U2-associated proteins.

Class	HDAC	Splicing-associated HDAC-interacting proteins
Class I	HDAC1	SF3A1, SFPQ, STRAP
	HDAC2	DDX20, SFPQ, SMN1, SNRNP70, SRRM2
	HDAC3	SF3B1
	HDAC8	
Class IIa	HDAC4	SF1
	HDAC5	DDX20, ADAR, DHX15, DHX9, EFTUD2, NOP2, PRPF19, PRPF39, PRPF8, SF1, SF3A1, SF3B1, SF3B2, SF3B3, SFPQ, SNRNP200, STRAP
	HDAC7	
	HDAC9	ADAR, EIF6, FIP1L1, NOP2
Class IIb	HDAC6	CPSF1, DDX19A, DDX41, PUF60, SF3A1, SRRM2
	HDAC10	
Class III	SIRT1	SART1
	SIRT2-5	
	SIRT6	EFTUD2, PRPF8, SNRNP200
	SIRT7	CPSF1, DDX20, DDX27, DHX15, DHX16, DHX30, DHX36, DHX37, DHX38, DHX8, DICER1, EIF6, GEMIN4, HEATR1, NOP2, PNN, PRPF40A, KHSRP, PRPF6, PRPF8, PUF60, PRPF4B, SART1, SF3A1, SF3B1, SF3B2, SFPQ, SNRNP200, SNRNP70, SON, SRPK1, SRRM1, SRRM2, SRSF3, SRSF5, U2SURP, UTP14A, UTP18, U2AF2, UTP6, WDR36, XAB2
Class IV	HDAC11	DDX20, DDX27, DICER1, DKC1, GEMIN2, GEMIN4, HEATR1, NOP2, NOP56, PNN, PPAN, PRPF6, RBM22, SMN1, SON, SF3A1, SF3B2, SRRM2, TFIP11, UTP6, UTP14A, UTP18, WDR33, WDR36, XAB2, YBX2, ZNF326

## HDACS AND RNA SPLICING IN HUMAN DISEASES

### RNA splicing and HDAC6 in cancer

Although we have only begun to understand the link between dysregulated alternative splicing in diseases, there is an unquestionable association between abnormal RNA splicing and cancer (99). Numerous genes that encode key regulatory proteins in the hallmarks of cancer are regulated by alternative splicing. These include genes such as *TERT, VEGFA (VEGF), RAC1* and *FGFR2*, which promote cell proliferation, angiogenesis, invasion, metastasis, and replicative immortality (100–112). Like p53, among these alternatively spliced cancer-associated genes are non-histone HDAC substrates (Table 2). However, while the link between alternative splicing and cancer is not well understood, their association with HDACs is even more arcane.

**Table 2.** Examples of cancer-associated non-histone substrates of HDACs. The RNA encoding these substrates are regulated by alternative splicing

Alternatively spliced non-histone HDAC substrate	HDAC
p53	HDAC1, SIRT1 (82–84)
STAT3	HDAC3 (152)
PPARA	SIRT1 (153)
CTTN	HDAC6 (154)
MLH1	HDAC6 (155)
MAX	Class I-IV (156)
PKM2	SIRT6 (157)
PFKFB3	Class III (158)
PTEN	HDAC6 (159)
HSP90	Class I-II (160)

SRSF2 (SFRS2) is an important splicing factor involved in spliceosome assembly, alternative splicing, and splice-site selection. Like other serine/arginine splicing factors, SRSF2 must be phosphorylated in order to interact with mRNA. It has been shown that the acetyltransferase TIP60 (KAT5) acetylates SRSF2, indirectly downregulates SRSF2 phosphorylation and causes a reduction in its protein level. However, the overexpression of HDAC6 opposes acetylation and increased SRSF2 at the protein level, without affecting its mRNA level. This suggests that SRSF2 acetylation leads to proteasomal degradation, though whether this is via a ubiquitin dependent or independent mechanism is unknown (113).

The dysregulation of splicing regulators has been shown to cause numerous abnormal alternative splicing events in cancers (114–122) and SRSF2 is no exception. SRSF2 is upregulated in hepatocellular carcinoma (HCC), with its expression being positively correlated with tumor grade and negatively correlated with patient survival time. It alters splicing events which contribute to the proliferative and pro-survival nature of tumor cells. The importance of a properly functioning SRSF2 protein can be seen in vivo where SRSF2 was silenced in mouse hepatocytes, leading to liver failure and death, while injection of SRSF2-silenced HCC cells into mice resulted in an inability to form tumors. The results of these experiments suggest that SRSF2 is not only crucial to normal liver development, but also to tumor growth. In vivo, cells treated with siRNA SRSF2 exhibited a decrease in growth and colony formation, while overexpression of SRSF2 had the opposite effects. Knockdown of SRSF2 in HCC cells resulted in alternatively spliced variants in cancer-related genes including exon inclusion of *GCH1, STK39* and *TERF1* (*TRBF1*). The knocking-down of these genes inhibited cell growth, while knocking-down of their short variants had no effect (123). This shows that changes in the concentration of splicing factors, plays an important role in regulating splicing events of many genes that can lead to a more tumorigenic state.

The role of HDAC6 in HCC is controversial, with one study showing that HDAC6 is upregulated in HCC (124), while another study shows that HDAC6 was upregulated in only 20% of their pool of primary HCCs. However, the latter study was correlated with a higher tumor grade, a greater number of tumors, and a greater extent of invasion and migration (125). Because SRSF2 is an HDAC6 substrate, it is reasonable to speculate that the upregulation of HDAC6 may cause an overall upregulation of SRSF2 which accumulates in cells inhibited by TIP60-mediated degradation. Hence, this higher than normal concentration of protein may potentially lead to a dysregulation of splicing events, and promote cancer.

### RNA splicing and HDACs in myotonic dystrophy and in spinal muscular atrophy

In addition to cancer, changes in alternative splicing can be seen in many diseases (126,127), and some of these changes consequently have been linked to HDACs (Table 3). While the exact link between HDACs and these diseases have not been well explored, some HDAC inhibitors have proven to be promising therapeutic agents. For instance, myotonic dystrophy (DM) is a rare autosomal dominant disease characterized by muscle loss and weakness. There are two forms of DM, both of which result in aberrant splicing of *CLCN1*, which encodes for chloride voltage-gated channels, important for proper muscle movement. More specifically, the splicing changes of *CLCN1* in DM type 1 is a result of the sequestration of MBNL1, an RNA binding protein which functions in mRNA splicing. Studies have shown that treatment of DM1 patient derived fibroblasts, with HDAC inhibitors vorinostat and ISOX, increased the protein expression of MBNL1 and rescued the mis-splicing of *CLCN1* (126).

**Table 3.** Diseases associated with dysregulated RNA splicing and related HDACs

Disease	Spliced Gene	HDAC
Familial Dysautonomia	*IKBKAP* (161)	HDAC6 (162)
Spinal Muscular Atrophy	*SMN1* (163–165)	Class I (136)
Hutchinson-Gilford Progeria Syndrome	*LMNA* (166)	Class I-II (167)
Myotonic Dystrophy	*CLCN1* (168)	Class II (169)
Dilated Cardiomyopathy	*LMNA* (170)	Class I (171)
Early-onset Parkinson's Disease	*PINK1* (172)	HDAC3 (173)
Retinitis Pigmentosa	*PRPF6* (174)	Class I (175)
Myelodysplastic Syndromes	*U2AF1* (176,177)	Class I-IV (178)
Amyotrophic Lateral Sclerosis	*TARDP*; *FUS* (179–181)	HDAC6 (182)
Hair Cell Degeneration and Deafness	*REST (* *145* *)*	HDAC1 & HDAC2 (145)

It is no surprise that diseases are accompanied by mutations that lead to aberrant splicing patterns. These mutations range from affecting splice sites to splicing regulators to core spliceosome components. Any of these changes could lead to detrimental results, causing developmental impairments and could even lead to death. This can be seen in spinal muscular atrophy (SMA), a recessively inherited neuromuscular disorder which affects the α-motor neurons in the anterior horn of the spinal cord. As one of the most prevalent recessively inherited disorders, with an incidence of 1:6000–1:10000, approximately half of all patients die during infancy. While SMA is primarily caused by a homozygous absence of the *SMN1* gene, the copy number of the *SMN2* gene dictates the disease's severity (128,129). This is because both genes encode the SMN protein, however to different extents. *SMN1* and *SMN2* are practically identical, differing only by a single nucleotide change in exon 7 where there is a C to T transition. This silent mutation disturbs the exonic splicing enhancer and causes the exclusion of exon 7 in approximately 90% of *SMN2* transcripts, generating only 10% full length *SMN2* transcripts. Skipping exon 7 results in a truncated and non-functioning protein (130).

SMN is a critical protein involved in axonal transport of RNA, snRNP biogenesis, and spliceosome assembly (131–134). The knocking out of *Smn* in mice led to embryonic lethality (135). It has been shown that over-expressing TRA2B (hTra2-beta1) in primary fibroblast cultures derived from SMA patients can restore splicing by promoting exon 7 inclusion. Treating these fibroblast cultures with valproic acid (VPA), an HDAC inhibitor, increased the levels of both SMN and TRA2B (131). Additional studies treated SMA cell cultures with sodium butyrate, phenylbutyrate, suberoylanilide hydroxamic acid, or M344, all of which are HDAC inhibitors, and found an increase in SMN protein levels (136–140). Interestingly, M344 could be a potential therapy candidate because of its ability to increase SMN protein levels 7-fold at low concentrations where toxicity is minimal (136). VPA and M344 do not upregulate full length SMN2 protein solely by restoring the correct splicing pattern (seen by an increase in FL-SMN2:Δ7SMN2 ratio), but also by promoting a general increase in gene transcription (131,136). Another group observed the restoration of exon 7 in *SMN2* transcripts in type II SMA patient fibroblasts, following curcumin treatment. This was accompanied by an increase in the mRNA transcript and nuclear *SRSF1* protein levels. *SRSF1* knockdown experiments reduced exon 7 inclusion, suggesting that it is an important splicing regulator of *SMN2*. The increased levels of *SMN2* transcripts with exon 7 inclusion, as well as the increase in *SRSF1* expression, was inhibited by TSA treatment. Although the exact connection between these factors is unknown, the data supports the idea that there is a deacetylation step necessary for exon 7 inclusion in *SMN2* transcripts (141). The outcomes of these works are encouraging and provide hope for the possibility of targeting alternative splicing using HDAC inhibitors as potential treatment for SMA (142,143).

### HDACs and alternative splicing-dependent regulation in deafness

RE1-Silencing Transcription factor (REST), also known as Neuron-Restrictive Silencer Factor (NRSF), is a transcriptional repressor involved in the repression of neural genes in non-neuronal cells. CoREST (RCOR1), a corepressor of REST, is a component of a multi-subunit repression complex that contains HDAC1 and HDAC2 (144). Although REST is commonly known to be regulated by transcriptional repression in differentiating neurons, alternative splicing is essential for REST regulation in the inner ear (145). REST is inactivated through alternative splicing of its pre-mRNA in both neurons and mechanosensory hair cells of the ear. In humans, an intronic C to G variant of *REST*, which prevents SRRM4-dependent inactivation of REST, is associated with progressive hearing loss. Proper splicing of exon 4 of *REST* mRNA requires SRRM4, and a mutation in the *REST* gene is associated with defective exon 4 splicing and deafness. Mice heterozygous for exon 4 deletion have no apparent CNS defects, but lose all mechanosensory hair cells in the ear and fail to respond to sound. In organ of Corti cultures, HDAC inhibitors prevented degeneration of outer hair cells and rescued the *REST* exon 4-deficient. More interestingly, hair cells and hearing of exon 4 KO mice were rescued by treatment with SAHA (Vorinostat), an FDA-approved HDAC inhibitor. Although it is not known at this time if the consequence is a direct or indirect effect of these drugs on alternative transcriptional regulators or additional cell signaling pathways, the ability of HDAC inhibitors to rescue hair cells and the hearing of *REST* exon 4 KO mice is most likely through interfering with HDAC1′s and HDAC2′s activities in REST-dependent gene repression. Thus, in this situation, HDACs may not directly regulate *REST* gene splicing or alter splicing machineries critical for *REST* splicing. Rather, HDACs and RNA splicing play an important role to regulate a common pathway, and that a splicing defect can be overcome by manipulating HDACs.

## KNOWLEDGE GAPS IN THE INTERCONNECTION OF HDACS, HISTONE MODIFICATIONS AND SPLICING

As discussed, there is a myriad of genes which undergo aberrant splicing that lead to disease developments. One example is that aberrant splicing can contribute to the hallmarks of cancer. These splicing events facilitate cancer progression by promoting metastasis, proliferation, cell survival, angiogenesis, and epithelial to mesenchymal transition (EMT). While some of these alternative splicing events drive the production of pro-tumorigenic variants such as the anti-apoptotic protein BCL-X (BCL2L1), others are driven by the overexpression of splicing factors. The most prominently studied case is SRSF1, which is upregulated in different human tumor types, and regulates the splicing of many genes encoding cancer-related proteins such as BIN1, which normally inhibits cMYC (MYC). However, SRSF1 leads to exon 12a inclusion on *BIN1*, producing a protein variant that is unable to interact with and inhibit cMYC (146). Although we know that defects in alternative splicing plays a role in cancer, there is a large gap of knowledge in understanding the direct cause or link between cancer and splicing. Growing technologies has allowed us to better explore splicing, but there is much left to discover in this exciting field.

Currently, there is overwhelming evidence that histone modifications and histone modifying enzymes play a role in the regulation of RNA splicing. Likewise, there is undoubtedly a link between splicing, acetylation/deacetylation and HDACs. However, a clear mechanistic association has yet to be determined for the splicing-HDACs connection. Further exploratory investigations as well as intensive confirmation studies are needed to determine if additional HDAC substrates that are splicing factors exist, and if some of the potential HDAC substrate/splicing regulators are in fact true non-histone substrates of HDACs. The potential interaction between HDACs and kinases that are important in phosphorylating and activating SR proteins, also need to be examined in more detail.

One of the most interesting findings in recent HDAC research is that some HDACs possess novel enzymatic activities that are much more efficient than their deacetylase activity. For example, HDAC8, HDAC11, SIRT2 and SIRT6 catalyze lysine defatty-acylation (42,147–149). Unlike histone deacetylation, at this time, we do not know if lysine defatty-acylation regulates gene transcription and consequently controls RNA splicing. Also unknown is whether any RNA splicing proteins are modified by fatty-acylation and regulated by HDACs via lysine defatty-acylation. Answers to these questions will be critical in obtaining a complete understanding of the HDAC-splicing connection and how HDACs regulate RNA splicing.

Another remarkable recent discovery in HDAC research is the finding that HDAC substrates extend beyond histone and non-histone proteins. For example, HDAC10 is a robust polyamine deacetylase (150). Polyamines are low molecular weight aliphatic polycations, ubiquitously present in all living cells. Naturally abundant polyamines include putrescine, spermidine, and spermine. Like HDACs and RNA splicing machineries, polyamines have many functions including regulation of chromatin structure, gene transcription and translation, signal transduction, cell growth and proliferation. Acetylation is a means to decrease the net positive charge of the polyamines and consequently release polyamines from anionic binding sites.

HDAC10 possesses optimal catalytic activity and specificity for the hydrolysis of *N*^8^-acetylspermidine into spermidine (150). Spermidine in turn is acetylated by spermidine/spermine acetyltransferase SSAT (SAT1), modifying it into *N*^1^-acetylspermidine. Interestingly, SSAT expression is controlled by RNA alternative splicing (151). During splicing, the intron between exon 3 and exon 4 of *SSAT*, which contains multiple stop codons might be retained. In the presence of high levels of polyamines, the formation of this alternative splice variant is decreased, leading to increased SSAT activity and therefore polyamine acetylation. Depletion of cellular spermidine promotes the exon inclusion and decay of *SSAT* transcripts. Thus, like protein p53 which is regulated by HDACs at the RNA splicing and the PTM levels, polyamine metabolism is also potentially regulated by HDACs and acetylation/deacetylation at multiple levels. The obvious question that remains is whether *N*^1^-acetylspermidine, like *N*^8^-acetylspermidine, can be deacetylated to spermidine; and if so, which HDAC(s) might be involved?

HDAC inhibitors have been extensively investigated and hold promises for the treatment of many diseases. Several hundred clinical trials involving HDAC inhibitors have been carried out or are ongoing, and five HDAC inhibitors are currently approved for treating cancer. Significant advances have been made in recent years in the development of approaches to manipulate and correct aberrant splicing for disease therapy. However, with the exception of SMA, little is known if HDAC inhibitors, either alone or in combination with other drugs, can be used to target RNA splicing and treat diseases associated with RNA splicing defects in general. The continuing development of more selective and highly potent HDAC inhibitors, coupled with a deeper understanding of the mechanisms and pathways by which HDACs regulate splicing, could pose powerful therapeutic strategies against many diseases due to aberrant RNA splicing.

## PERSPECTIVES

Histone modifications and RNA splicing regulating gene expression are startling independent discoveries of the 1960s and 1970s that profoundly changed the way we think about, and approach the study of gene expression. The recent finding of a potential HDAC-splicing connection is unexpected and represents yet another unique and exciting avenue of research to obtain a greater understanding and deeper appreciation of the complexity of eukaryotic gene expression. Some of the initial HDAC-splicing connections were made with the use of data derived from genome-wide chromatin immunoprecipitation and SILAC studies. As increasingly sophisticated techniques to study gene regulation become available, we predict the link between HDACs and splicing will become clearer and there will undoubtedly be more histone/protein modifying enzymes that can be shown to play a role in RNA splicing. Current work in many laboratories are focused on understanding the biological and physiological relevance of the HDAC-splicing interconnection and identification of additional histone modifying enzymes that regulate RNA splicing. With some HDAC inhibitors already being clinically used to treat diseases, it is safe to predict that targeting HDACs to potentially overcome diseases related to splicing-defect holds great promise.
